# Assembl y of Poly-3-Hexylthiophene Nano-Crystallites into Low Dimensional Structures Using Indandione Derivatives

**DOI:** 10.3390/nano3010107

**Published:** 2013-02-01

**Authors:** Nicolas Cheval, Valdis Kampars, Clifford Fowkes, Neil Shirtcliffe, Amir Fahmi

**Affiliations:** 1 Department of Mechanical, Materials and Manufacturing Engineering, University of Nottingham, Nottingham NG7 2RD, UK; E-Mails: lop3ks@googlemail.com (N.C.); sevendof@gmail.com (C.F.); 2 Faculty of Materials Science and Applied Chemistry, Riga Technical University, Riga, LV 1048, Latvia; E-Mail: kampars@ktf.rtu.lv; 3 Faculty of Technology and Bionics, Rhein-Waal University of Applied Sciences, Marie-Curie-Straße 1, D-47533 Kleve, Germany; E-Mail: neil.shirtcliffe@hochschule-rhein-waal.de

**Keywords:** poly-3-hexylthiophene, directed self-assembly, indandione derivatives, nanofibers

## Abstract

Conductive polymer poly-3-hexylthiophene (P3HT) needles were self-assembled using a second component (indandione derivatives) as a linking agent to enhance their long range alignment. The morphologies of the hybrid organic/organic materials were characterized by transmission electron microscopy (TEM). Both linear and branched structures could be produced, with the degree of branching depending upon the linker used. Incorporation of indandione derivatives broadened the UV absorbance band of P3HT without significant change to its photoluminescence. This hybrid material could open a promising avenue in photovoltaic applications due to its interesting morphologies and optical properties.

## 1. Introduction

Since the discovery of the photovoltaic (PV) effect by the French physicist Edmond Becquerel in 1839, many materials have been investigated for their PV properties. More recently, conjugated polymers such as Poly-3-hexylthiophene (P3HT) have received considerable interest for use in polymer solar cell (PSC) fields due to their processability, flexibility and excellent electrical properties [1,2,3,4,5].

It has been widely reported that the electrical performance of P3HT depends strongly on its orientation, morphology and also on its crystal structure [6,7,8]. An ordered P3HT crystal structure facilitates efficient electron transport [9].

Recent developments have shown that modified P3HT with inorganic moieties [10] and homopolymer provides a simple and low-cost method to fabricate nanostructured materials for optoelectronic applications [11,12]. For instance, Su *et al.* enhanced the performance of photovoltaic device by blending (P3HT) with polymethylmethacrylate (PMMA) [13].

Herein a simple and inexpensive method is described that can be used to fabricate well-defined structures materials by blending P3HT with Indandione derivatives. These were co-precipitated with P3HT to study the properties of the mixed system. Two types of dimethylaminobenzylidene-1,3-indandione (DMABI) derivatives (DMABI-Ju and DMABI-OH) were used [14].

Dimethylamininobenzyllidene-1-3-indandione (DMABI) is a class of polar organic derivatives of 1,3-indandione composed of a chromophoric electron donor fragment (DMAB) and a chromophoric electron acceptor fragment connected by the –CH= bridge [15,16]. Due to this asymmetric charge distribution and the conjugated π-system, this organic dipolar molecule possesses excellent optical, photoluminescence and electrical properties [17,18,19]. For this reason it is a suitable active addition to P3HT.

## 2. Methods

### 2.1. Synthesis

Chemicals were purchased from Sigma-Aldrich and used as supplied. DMABI derivatives were prepared in Riga Technical University by the second author using the method described in a previous publication [14].

Regioregular Poly-3-hexylthiophene-2,5-diyl (P3HT) (regioregularity > 90% and Mw = 45.000 g/mol) was purchased from Sigma Aldrich and used as received. The chemical unit of P3HT is (C10H14S)n and its molar weight is 166.28 g mol^−1^.

P3HT and the indandione derivatives were dissolved in separate portions of THF with the aid of an ultrasonic bath. A stoichiometric amount of one of the indandione derivatives was added to the P3HT solution to yield a molar ratio between the monomers of the materials of 1:1. The mixture was stirred for 24 h to ensure the coordination between the materials. The solvent was allowed to evaporate at room temperature and the resulting solid collected in an evaporating dish.

### 2.2. Measurements

TEM measurements were performed using a TECNAI Biotwin (FEI Ltd., Valley City, ND, USA) at 100 keV to investigate the morphology of P3HT and P3HT/indandione derivative system. The instrument was operated at low beam intensities to prevent electron damage of the polymer samples. P3HT and P3HT/indandione derivative solutions were deposited on carbon-coated copper grids (400 mesh, AGAR Scientific, London, UK) and dried at room temperature. UV-Vis absorption spectra of P3HT and P3HT/indandione derivative system were obtained in solution at room temperature using Varian Cary 50 from Varian Inc. (Palo Alto, CA, USA). Photoluminescence measurement was carried out at room temperature in solution using Varian Eclipse photospectrometer. Electrical measurements were performed with a potentiometer. The sample was prepared by deposing the solution on a glass substrate of 7 mm of width and 20 mm length to form a homogenous film. Thermal properties of P3HT were determined using a DSC model Q 10 (TA Instrument, New Castle, PA, USA) under nitrogen between 40 °C and 280 °C. The sample was firstly heated at 280 °C for 5 min to erase the thermal history of the polymer. Then, the samples were cooled to 40 °C and heated to 280 °C at 10 °C/min.

Attenuated total reflection IR (ATR-FTIR) spectrometry was performed to identify the type of interaction between P3HT and indandione derivatives. The measurements were carried out on a thin polymer film formed after evaporation of the solvent on the ATR crystal.

## 3. Results and Discussion

**Figure 1:**
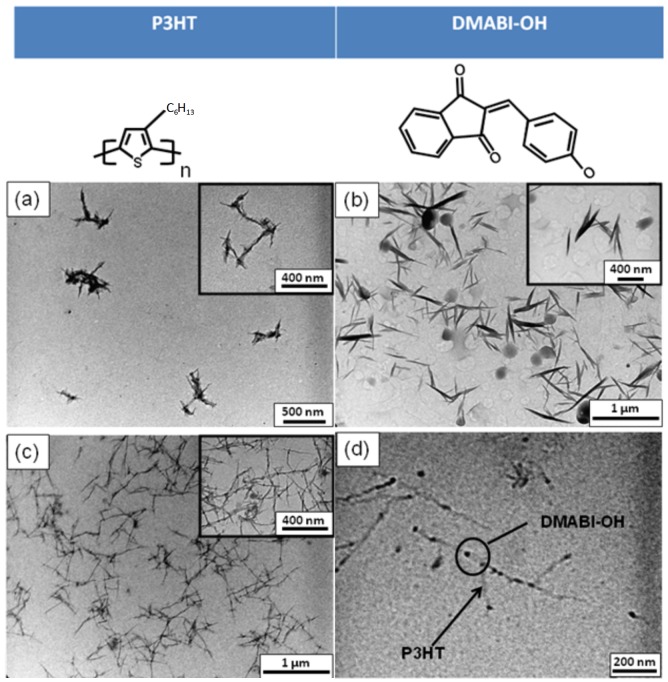
TEM Images of structures formed with P3HT and DMABI-OH (**a**) P3HT nano-crystals from THF; (**b**) DMABI-OH crystals from THF (**c**); and (**d**) structures from mixed solutions of DMABI-OH and P3HT. The difference in contrast along the nanofibers is attributed to the differences of the DMABI-OH concentration along the nanofibers; the darker contrast reflects the richer DMABI-OH constituencies.

**Figure 2:**
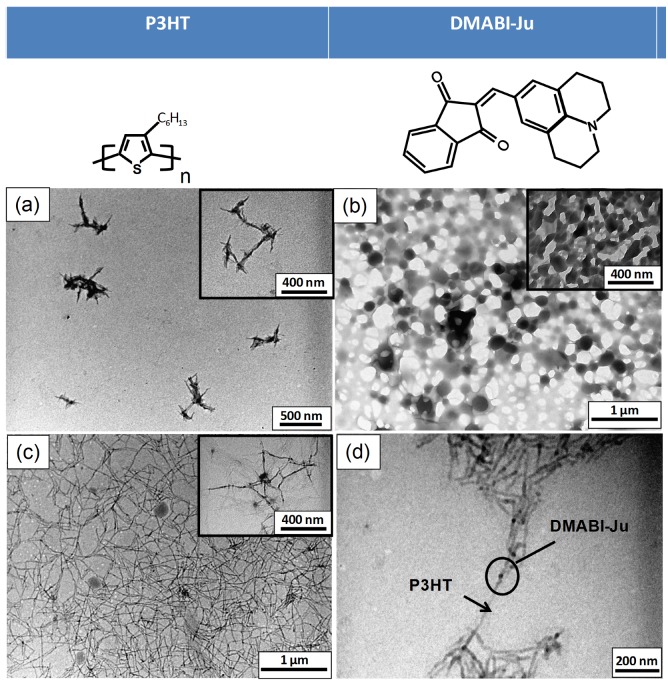
TEM Images of structures formed with P3HT and DMABI-Ju (**a**) P3HT nano-crystals from THF; (**b**) DMABI-Ju crystals from THF (**c**); and (**d**) structures from mixed solutions of DMABI-Ju and P3HT. The difference in contrast along the nanofibers is attributed to the differences of the DMABI-Ju concentration along the nanofibers; the lighter contrast reflects the richer P3HT constituencies.

Figure 1a and Figure 2a are TEM images showing aggregates of short P3HT crystalline nanoneedles. As P3HT is a semi-crystalline polymer (Supplementary Information) the formation of P3HT lamellar structure depends not only on the processing condition but also on the interaction between the polymer and the solvent. Yang *et al.* reported that the P3HT crystalline lamellae can be obtained in various solvents including toluene, chloroform and THF [9]. In comparison DMABI-OH forms comparatively thick needles (Figure 2b) and DMABI-Ju, larger, round crystals.

Combining P3HT and the indandione derivatives led to the self-assembly of an extended nanofiber network (Figure 1c, Figure 2c). Linear features linked with dark dots can be seen on the micrographs. The difference of contrasts between these two areas reveals that the nanofibers are probably composed of two phases. The lighter linear features are probably rich in P3HT and the darker connections rich in DMABI-Ju. The same black dots are also observed for P3HT/DMABI-OH, although in this case more branched structures were formed and the extent of the network was lower. This significant contrast is attributed to the differences of the indandione derivatives concentration along the nanofibers; in the light portions one of the DMABI-Ju/ DMABI-OH links two P3HT chains through mainly H-bonds. Nevertheless, dense numbers of DMABI-Ju/ DMABI-OH aggregations are involved with different types of interactions to link the P3HT chains within the dark portions along the nanofibers. This suggests that the indandione derivatives could be used as a linker to assemble the P3HT crystalline nanoneedles into high aspect ratio of hybrid nanofibers. These possess uncontrolled heterogeneous aggregations of the indandione derivatives assembled along the unidirectional hybrid nanostructures.

### 3.1. ATR-FTIR Analysis

**Figure 3:**
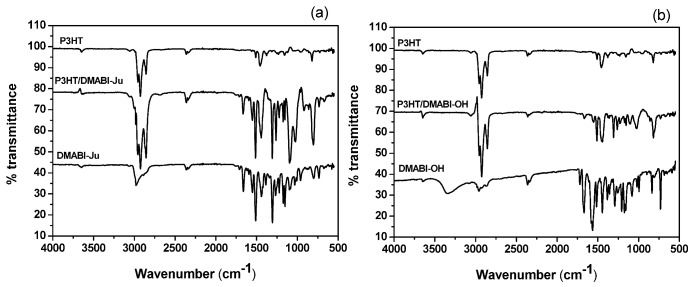
(**a**) ATR IR spectra of P3HT/DMABI-Ju; (**b**) ATR IR spectra of P3HT/DMABI-OH.

To investigate the interaction between P3HT and the indandione derivatives, attenuated total reflection IR measurements were conducted (Figure 3). These show that carbonyl absorption band of the DMABI derivatives is shifted in the presence of P3HT, indicating that the two materials interact and the carbonyl bond is involved in some way, either directly or through the π system. DMABI-OH monomer contains two carbonyl groups and an alcohol group (–OH). As reported by Soscuan *et al.*, a thiophene group can also interact with a hydroxyl group (–OH) through van der Waals interactions [20]. As can be seen the –O–H stretch band of the DMABI-OH around 3400 cm^−1^ is not present in the mixture (Table 1), suggesting that H-bonded complexes of bridging OHs are not only formed with sulphur, but also through the π-type electron cloud of the thiophene ring to increase the perturbation of the OHs [21,22,23].

**Table 1:** Absorption maxima of the carbonyl group of DMABI in the various systems investigated here.

Sample	C=O stretch
DMABI-OH	1669.12 cm^−1^
P3HT- DMABI-OH	1666.97 cm^−1^
DMABI-Ju	1662.34 cm^−1^
P3HT- DMABI-Ju	1665.25 cm^−1^

### 3.2. Optical Spectroscopy

A polymer based photovoltaic device performance depends strongly on the light absorption by the conducting polymer to convert the photo energy into free electrons [24].

**Figure 4:**
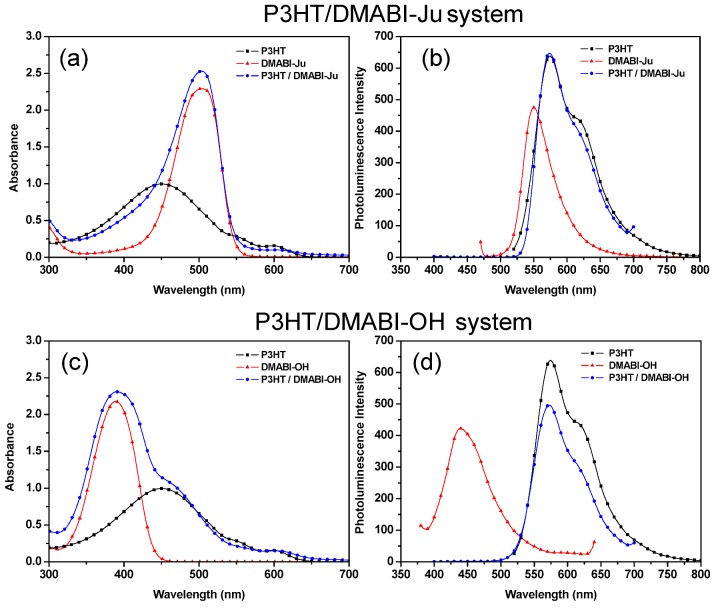
(**a**) UV-Vis spectra of P3HT/DMABI-Ju; (**b**) UV-Vis spectra of P3HT/DMABI-OH; (**c**) Fluorescence spectra of P3HT/DMABI-Ju; (**d**) Fluorescence spectra of P3HT/DMABI-OH.

UV-Vis spectrometry was performed to investigate the optical properties of the extended structures and pure P3HT as well as P3HT/DMABI-Ju system in solution. (Figure 4a,c) The maximum absorption wavelength at around 450 nm corresponds to the π-π transition of P3HT [25]. DMABI-Ju exhibits a maximal wavelength at 500 nm and DMABI-OH at approximately 370 nm. The mixture of P3HT and the indandione derivatives cover a larger absorption range than the neat homopolymer. The absorbtion peak of P3HT and DMABI-Ju was extended to the red, while DMABI-OH extends the absorption range of P3HT to the blue.

Photolumiscence spectrometry was carried out to study the possible charge transfer occurring between P3HT and indandione derivatives (Figure 4b,d). The P3HT peak is virtually unchanged in the presence of the DMABI monomers but the luminescence band of both of the DMABI groups is completely absent. This could be due to energy transfer or charge transfer between the molecules [26].

### 3.3. Electrical Behavior Under Illumination

**Figure 5:**
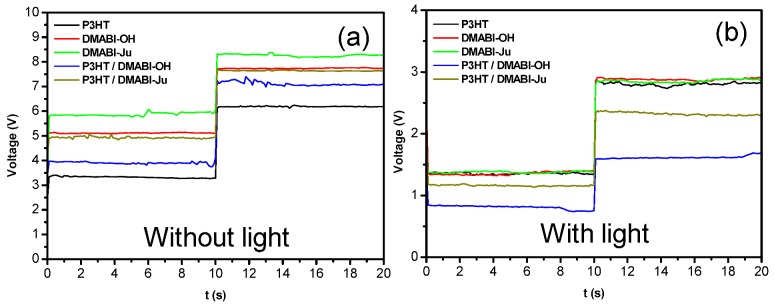
Plots of voltage *versus* time of P3HT, DMABI-OH, DMABI-Ju, P3HT/DMABI-OH system and P3HT/DMABI-Ju system without light (**a**) and with exposure to light (**b**) for a current at 1×10^−8^ A stepping up to 2×10^−8^A.

In order to investigate the potential of these novel hybrid materials for solar cell applications, a measurement of the voltage *versus* time at constant current (1 × 10^−8^ A and 2 × 10^−8^ A) was performed on a thin film of P3HT, DMABI-OH, DMABI-Ju, P3HT/ DMABI-OH system and P3HT/DMABI-Ju system in the light and in the dark (Figure 5). This simple test provides some information about the electrical behavior of these materials since the voltage is related to the resistivity and conductivity. The results show that all materials are conductive and light sensitive (Figure 5). Nevertheless, incorporation of indandione derivative affects the P3HT electrical behavior. For instance, without exposure to light, the resistivity (R) of P3HT/indandione derivative hybrid material increases. However, under light, the presence of DMABI monomers increases the P3HT conductivity. The difference of conductivity observed between the two hybrid materials under light could be related to their optical performances since these two systems do not absorb at the same wavelength.

## 4. Conclusions

A simple method has been proposed to self-assemble P3HT nanocrystals using indandione derivatives. Controlling the physical interaction between P3HT and the indandione derivatives is one of the key factors to guide and assemble the P3HT crystalline lamellae. The indandione derivative is used as a linker to build linear and branched nanowires. Extension of the P3HT absorption spectra in the IR or UV region due to the presence of the linker will enhance the performances of the photovoltaic devices since the system absorbs over a larger spectral range. These hybrid materials could find application as a photovoltaic material due to their interesting morphologies and optical properties.

## Supplementary Files

Supplementary File 1
