# Supplementary material for: Assembl y of Poly-3-Hexylthiophene Nano-Crystallites into Low Dimensional Structures Using Indandione Derivatives
Source: Nanomaterials (Basel). 2013 Feb 1;3(1):107–16. doi: 10.3390/nano3010107 (PMC5304924; doi:10.3390/nano3010107)
Supplement: Supplementary File 1 [file nanomaterials-03-00107-s001.doc]

*Nanomaterials* **2013**, *3*, 107-116; doi:10.3390/nano3010107

***§***

**OPEN ACCESS**

***nanomaterials***

**ISSN 2079-4991**

www.mdpi.com/journal/nanomaterials

Article

Assembly of Poly-3-Hexylthiophene Nano-Crystallites into Low Dimensional Structures Using Indandione Derivatives

Nicolas Cheval ^1^, Valdis Kampars ^2^, Clifford Fowkes ^1^, Neil Shirtcliffe ^3^ and Amir Fahmi ^1,3^,*

^1^ Department of Mechanical, Materials and Manufacturing Engineering, University of Nottingham, NG7 2RD, UK; E-Mails: lop3ks@googlemail.com (N.C.); sevendof@gmail.com (C.F.)

^2^ Faculty of Materials Science and Applied Chemistry, Riga Technical University, Riga, LV 1048, Latvia; E-Mail: kampars@ktf.rtu.lv

^3^ Faculty of Technology and Bionics, Rhein-Waal University of Applied Sciences,
Marie-Curie-Straße 1, D-47533 Kleve, Germany;
E-Mail: neil.shirtcliffe@hochschule-rhein-waal.de

* Author to whom correspondence should be addressed;
E-Mail: amir.fahmi@hochschule-rhein-waal.de; Tel.: +49-2821-806-73-634;
Fax: +49-2821-806-73-163.

**Supplementary Information**

**1. Synthesis**

P3HT and indandione derivatives were separately dissolved in THF solvent. Ultrasonic bath was used to help their dissolution. A stoichiometric amount of indandione derivative was added to the P3HT solution to yield a molar ratio between both materials of 1:1. The mixture was stirred for 24 h to ensure the coordination between both materials.

**2. Measurements**

TEM measurements were performed using a TECNAI Biotwin (FEI Ltd.) at 100 keV to investigate the morphology of P3HT and P3HT/indandione derivative system. The instrument was operated at low beam intensities to prevent electron damage of the polymer samples. P3HT and P3HT/indandione derivative solutions were deposited on carbon-coated cupper grids (400 meshes, AGAR Scientific) and dried at room temperature. UV-Vis absorption spectrum of P3HT and P3HT/indandione derivative system was obtained in solution at room temperature using Varian Cary 50 from Varian Inc. Photoluminescence measurement was carried out at room temperature in solution using Varian Eclipse photospectrometer. Electrical measurements were performed with a potentiometer. The sample was prepared by depositing the solution on a glass substrate of 7 mm of width and 20 mm length to form a homogenous film. A lamp of 40 Watt was placed at 4 cm of the film and the conductivity of each film was measured for 10 s.

Thermal properties of P3HT were determined using a DSC model Q 10 (TA Instrument) under nitrogen atmosphere in the temperature range between 40 °C and 280 °C. The sample was firstly heated at 280 °C for 5 min to erase the thermal history of the polymer. Then, the samples were cooled to 40 °C and heated to 280 °C at 10 °C /min.

**3. Results and Discussion**

*DSC Analysis*

To support the notion that P3HT is a semi-crystalline thermoplastics polymer, DSC measurement was performed at 10 °C /min. (Figure S1) The melting temperature has been determined at 224 °C and the crystallisation temperature at 191 °C. The exothermal peak observed confirms that P3HT is a
semi-crystalline polymer.

**Figure S1.** DSC melting and crystallisation thermograph of P3HT at 10 °C/min.

© 2013 by the authors; licensee MDPI, Basel, Switzerland. This article is an open access article distributed under the terms and conditions of the Creative Commons Attribution license (http://creativecommons.org/licenses/by/3.0/).
